# Young people's moral attitudes and motivations towards direct-to-consumer genetic testing for inherited risk of Alzheimer disease

**DOI:** 10.1016/j.ejmg.2021.104180

**Published:** 2021-06

**Authors:** Gabriela Pavarini, Lamis Hamdi, Jessica Lorimer, Ilina Singh

**Affiliations:** a Department of Psychiatry, University of Oxford, UK; b Wellcome Centre for Ethics and Humanities, University of Oxford, UK

**Keywords:** Predictive testing, APOE, Alzheimer disease, Genetic testing, Young people, Ethics, Consent

## Abstract

**Purpose:**

Since the U.S. Food and Drug Administration approved sales of genetic tests for late-onset Alzheimer's disease (LOAD) risk, a heated debate has arisen over whether these tests should indeed be offered online and direct-to-consumer (DTC). As this debate progresses, it is important to understand the ethical perspectives and motivations of young people, who are a key target group for DTC services.

**Methods:**

Thirty-one grandchildren of people with LOAD, aged 16–26, were interviewed about their moral attitudes and motivations with regards to DTC genetic testing for LOAD.

**Results:**

Even though most participants claimed that people should have the right to access these services, they also expressed concerns about potential distress in response to learning about risk, particularly for minors. About a third were interested in testing, primarily to gain self-knowledge regarding one's health; however, face-to-face services were vastly preferred over the online option.

**Conclusion:**

While DTC genetic companies often market their services as a “fun consumer product”, DTC testing for LOAD was largely understood as a serious health screening procedure and a vulnerable moment in the lives of young people in Alzheimer's families. This points to the importance of appropriate standards of information and support to young people pre- and post-testing.

## 1 Introduction

In April 2017, the U.S. Food and Drug Administration (FDA) allowed the genetic testing company 23andMe to offer direct-to-consumer (DTC) testing for late-onset Alzheimer disease (LOAD) risk (FDA, 2017). Since then, the service has been made available to several other countries including the UK, Ireland and Denmark. The test analyses a gene called APOE, which is responsible for the metabolism and transportation of LDL cholesterol and other lipids, and has three major allelic variants: ε2, ε3 and ε4. APOE ε4 is associated with an increased risk for LOAD, whereas APOE ε2 is mildly protective (Corder et al., 1993; Suri et al., 2013). Variations in the APOE gene are, however, only one among many factors that influence a person's risk, and the presence of APOE ε4 is neither necessary nor sufficient for developing the condition (Henderson et al., 1995). Due to its low specificity and predictive value, as well as the lack of preventative measures for LOAD, predictive genetic testing for APOE is not currently offered by the public health system and is not recommended by professional bodies (Goldman et al., 2011).

The lifting of FDA restrictions and increasing international availability of DTC tests brought about a considerable influx of journal articles and grey literature/blogs discussing the harms and benefits of offering this test directly to the consumer (Lucassen, 2015; Zallen, 2018; Rohn, 2018). On the bright side, scholars have argued that DTC tests promote the democratisation of medicine and increase people's sense of agency (Allyse et al., 2018; Vayena, 2015). Knowing one's risk can inspire positive health behaviours (e.g. setting health priorities, being vigilant about early symptoms) (Marshe et al., 2019). On the other hand, providing risk results in the absence of healthcare professionals might put individuals at risk of distress and anxiety, particularly if they misinterpret the value and utility of the results (Zallen, 2018; Marshe et al., 2019). Concerns surrounding privacy and protection of data obtained by DTC companies have also been raised (Caulfield and McGuire, 2012). Largely due to these concerns, DTC genetic testing for disease susceptibility without genetic counselling is currently restricted or banned in a number of European countries including Germany, Spain and France (Kalokairinou et al., 2018).

Given that DTC tests are heavily marketed to young generations (Felzmann, 2015), an equally relevant debate concerns whether DTC tests for LOAD should be accessed by young people before the age of majority (in most countries regarded as 18 years). Most professional guidelines including the European Society for Human Genetics and the American Academy of Paediatrics advise against predictive genetic testing of minors for late-onset disorders, unless opportunities for prevention and early intervention are available (Borry et al., 2006; Garrett et al., 2019). This is largely aimed at safeguarding the child's “right to an open future” (Borry et al., 2014; Mand et al., 2012). Deferring the test also aims at minimising potential psychosocial harms of learning about one's risk of disease (Wade et al., 2013). Some guidelines, however, suggest exceptions might be valid, for instance when testing alleviates disabling parental anxiety (Ross et al., 2013).

Despite current guidelines, a majority of DTC testing companies do perform genetic testing in under 18s (upon parental request) and mechanisms allowing minors to voice their assent are scant (Borry et al., 2009). With regards to DTC testing for LOAD, in particular, 23andMe declares that the service is designed for adults over the age of 18, but states that any parent willing to open an account and test their child is welcome to do so, without providing further guidance (23andMe, 2020). There are no stringent conditions for testing a minor, and no criteria that minors must meet to be tested for certain conditions. Moreover, even though the Terms and Conditions require that users provide true and accurate information, there is no clear way to validate a user's age. In sum, there is an evident dissonance between what the professional guidelines recommend and what these external services offer.

As these ethical discussions – surrounding both the general permissibility of DTC genetic testing for LOAD and the acceptability of offering such tests to minors – progress, we argue that the voices of young people have been noticeably absent. Yet, adolescents and emerging adults are a key target of DTC tests advertising efforts (which rely heavily on social media and digital influencers (Kalokairinou et al., 2017)), making them key stakeholders in the debate.

Even though we know very little about their moral attitudes and motivations towards DTC tests for LOAD, there is a growing literature on young people's attitudes towards general DTC tests (e.g., ancestry + health package). The existing studies, so far exclusively survey-based, indicate that University students express a high interest in taking such tests, largely motivated by a desire to contribute to scientific research (Giraldi et al., 2016; Vayena et al., 2014),. However, they also express concerns surrounding privacy and potential implications for health insurance (Mackert et al., 2012). We currently lack evidence on young people's level of interest in and motivating factors for LOAD testing in particular, and on their attitudes regarding whether this test should be available online and DTC.

Furthermore, no previous research has investigated young people's attitudes on whether such tests should be accessible to children and adolescents. Studies outside the DTC context suggest that under-18s feel stressed and disempowered when their requests to know their risk status for physical conditions such as Huntington's Disease and breast cancer are not supported (Mand et al., 2013; Duncan et al., 2008). However, in a different study, young people argued that minors, who are still “finding [their] way in life”, may not be ready to receive bad news about their genetic makeup (Macleod et al., 2014).

These results may or may not map onto attitudes to APOE testing in the DTC context. Notably, APOE is a different case to the conditions mentioned above. It has low predictive value for late-onset AD, and poor clinical utility, in comparison to single-gene disorders and other disorders for which genomic markers have more predictive power and for which there are evidence-based treatments. Moreover, LOAD testing is arguably different from other DTC tests such as ancestry, which more naturally fall under the umbrella of “recreational genomics” (Farkas and Holland, 2009; Evans, 2008).

The present study investigates the following main questions:

1. What are young people's moral attitudes regarding online DTC genetic testing for LOAD?
2. What are their ethical perspectives on whether minors should be allowed to access such services?
3. What are young people's motivations for taking (or not taking) a test for LOAD?

We examined these questions via qualitative interviews with a sample of young grandchildren of people with LOAD. Grandchildren often have experiential knowledge about the condition (Celdrán et al., 2011), and predictive genetic testing is potentially especially relevant for this population. We recruited young people aged 16–26, which largely represent adolescents but also young adults, following the expanded definition proposed by Sawyer and colleagues (Sawyer et al., 2018).^1^ In addition to being a target market for DTC tests, this age group has just recently reached (or will soon reach) the age of majority and therefore could offer valuable insights into the ethics of testing minors.

## 2 Materials and methods

### 2.1 Participants and study design

Participants were recruited from the United Kingdom, particularly Oxfordshire and Greater London areas, through word-of-mouth, and online school and university noticeboards. Inclusion criteria included having a biological grandparent diagnosed with LOAD and being aged 16–26. One-to-one, semi-structured interviews of about 1 h were conducted by one of three female interviewers (LH, GP or JL) at a university location. Interviews were digitally recorded and transcribed in full. Approval for the study was given by the University of Oxford Central University Research Ethics Committee (R55781/RE001), and written consent was obtained at the start of the interview.

### 2.2 Interview guide

The interview guide was co-designed with the University of Oxford Neuroscience, Ethics and Society Young People's Advisory Group (NeurOX YPAG). This is a group of young people between the ages of 15 and 18 who have worked with our team as co-actors in this research, and across several projects related to ethics and predictive psychiatry. This helped ensure that our questions and methods were relevant and appropriate for the target group (Pavarini et al., 2019).

**Fig. 1:**
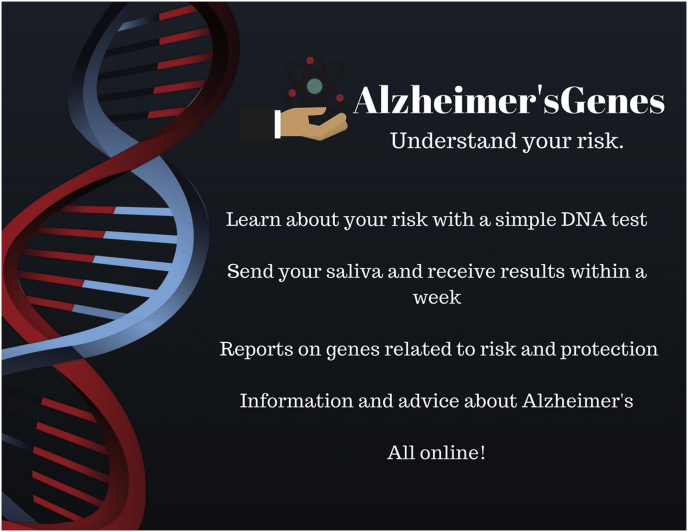
Fictional advertisement from a DTC testing company for LOAD risk.

The interviews were divided in two sections. The first covered participants' relationships with their LOAD-diagnosed grandparent and the impact of LOAD on family relationships (results from this section are not included in the present paper). The second section covered participants’ values and preferences regarding predictive testing for LOAD. At the start of this section, participants watched a video clip about the genetics of Alzheimer disease (available at https://www.youtube.com/watch?v=kFno-K6ybS8). This video offered information about the disease and clarified that LOAD is a complex, multi-determined condition, and that predictive genetic testing offers a probability and not a definite result. Participants were then asked to respond to a fictional advertisement from a company claiming to give information about LOAD risk (Fig. 1), which served as a prompt to initiate a conversation about DTC genetic testing. The video and advert were meant to support young people, who may lack knowledge about the genetics of LOAD, to express their perspectives based on realistic information about what a predictive test might be able to offer, and more confidently engage in dialogue about the ethics of DTC services. Relevant questions from this section are outlined in Box 1. At the end of the interview, participants also filled in a short demographic form.

Box 1 **Sample interview questions regarding predictive testing for LOAD risk**

**Figure:**
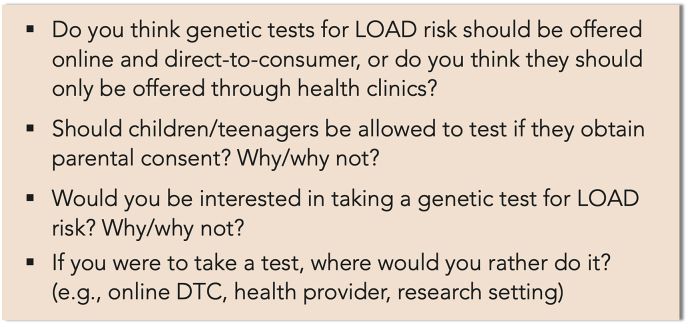

Alt-text: Box 1

### 2.3 Data analysis

Anonymised transcripts were coded using thematic analysis by LH, GP and JL (Braun and Clarke, 2006). All transcripts were used to inductively develop the initial coding frame, which identified the major themes and subcategories. This coding frame was drawn up by two coders and further validated by members of the NeurOX YPAG. Following the final coding scheme, each transcript was independently coded by two investigators to ensure that codes were consistent and transparent, reaching satisfactory inter-rater reliability (κ > 0.81 for all coding schemes).

## 3 Results

### 3.1 Participants

Thirty-one participants aged 16–26 were interviewed (*M*_age_ = 20.9; *SD*_*age*_ = 2.1). There were 21 women and 10 men. All participants were in full-time education and had a biological grandparent with a LOAD diagnosis. Some participants had caring responsibilities for their grandparent, either at the time of the interview or previously (for example, if the grandparent had already passed away), while others had only sporadic contact.

### 3.2 Moral attitudes towards DTC testing for LOAD

**Fig. 2:**
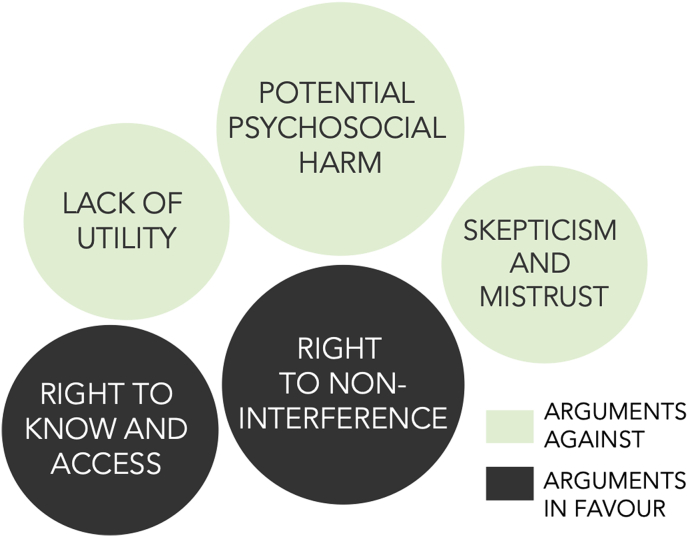
Core arguments for and against DTC testing for LOAD risk; larger circles represent more prominent themes.

When describing their attitudes regarding whether genetic testing for LOAD should be offered online and DTC, about two thirds agreed for the test to be offered, whereas a third were against it. The core themes that emerged during this section of the interview are illustrated in Fig. 2. As shown, arguments for DTC testing were primarily motivated by autonomy values, in particular the right of non-interference i.e. the notion that users should have “free choice” (“if you *want* it, that's fine, just go for it”, Mary, 22) and the right to “decide for themselves what is helpful for them” (Amy, 21). Others argued that people have the “right to know” and to access information about their health, even when the significance of the information is uncertain.

Of course, if it [the test] exists and people want to know—because there are probably people that want to know—then this is their right. (Nina, 23).

I think it's important to make sure people can access the tools they need to know whether they are healthy or unhealthy. (Lily, 19).

Despite these generally liberal inclinations, most participants also expressed doubts and worries regarding DTC tests. About half expressed concerns around the lack of support and advice offered by DTC companies. This lack of support was the core reason why some thought online DTC tests for AD are not permissible, or only permissible if sufficient systems are in place to support users. Without explicit support, participants were concerned that consumers would experience psychosocial harm.

I think there should definitely be some sort of professional intervention and explanation to the person who's going to get the results and where they can go from there and that sort of support afterwards. But then I would just be tempted to say why not cut out the private companies and the online things and just let it come from professionals in the first place? (Maya, 19).

When discussing permissibility, about a third expressed concerns around the utility of these tests, given its low predictive value and the lack of effective prevention and early-intervention strategies for LOAD.

They're not going to tell you anything that's going to make you be able to change your lifestyle in a way that would stave it off (Antonio, 20).

Finally, many participants touched upon broader societal harms, including a fear of being exploited by DNA companies, and a general lack of trust in the service or the results that DTC companies might provide (*I'm quite sceptical of those [companies], in general, Edward, 20).* Some understood online DTC tests to be at odds with what they perceived to be the *medical* nature of the procedure and argued that it is “not something just for fun.”

Anyone can offer anything online. So it's hard to know whether it's trustworthy or not.

[It is] sort of exploitative (…) They're sort of playing on the deep emotions that Alzheimer's evokes in people who have known somebody who was diagnosed or, you know, had to deal with that loss (Lily, 19).

### 3.3 Moral attitudes towards testing of minors

There was little consensus when it comes to participants' attitudes on whether under 18s should be allowed to get tested (subject to parental consent). Participants offered a number of different arguments against and in favour of testing minors, which are summarised in Table 1. Here a similar ethical tension emerged, between the need to protect young people from harm and the need to respect their autonomy or that of their parents. With regards to the former, a major concern was that risk information might pose a risk to a minor's wellbeing and self-perception. The lack of practical utility of testing at such young age also emerged here a concern. On the other hand, those who agreed with this testing largely argued that it was the parents' right to decide, and a minority argued that adolescents should have the right to test when they are mature enough, regardless of their nominal age.

**Table 1:** Core arguments presented against and in favour of genetic testing for LOAD risk in minors.

**Arguments against**	**Example quote**
Potential harm to children and adolescents' wellbeing and self-identity	*I don't think it would be good for their emotional wellbeing to be thinking about that sort of thing really at that age (Antonio, 20).* *I would hate for it to impact what they think of themselves (Lily 19).*
Lack of practical benefit	*It's not going to affect anyone in the first 18 years of their life, so why? There's nothing preventative that you can do. (Lea, 18).*
**Arguments in favour**	**Example quote**
Parental rights	*I guess if their parents agree I can't really stop them (Amy, 21).*
Young people's rights	*It feels quite unfair when you're … 15 or so and you think you're completely … mature and able to deal with things and people don't just trust you (Kendra, 21).*

### 3.4 Motivations for genetic testing for LOAD

**Fig. 3:**
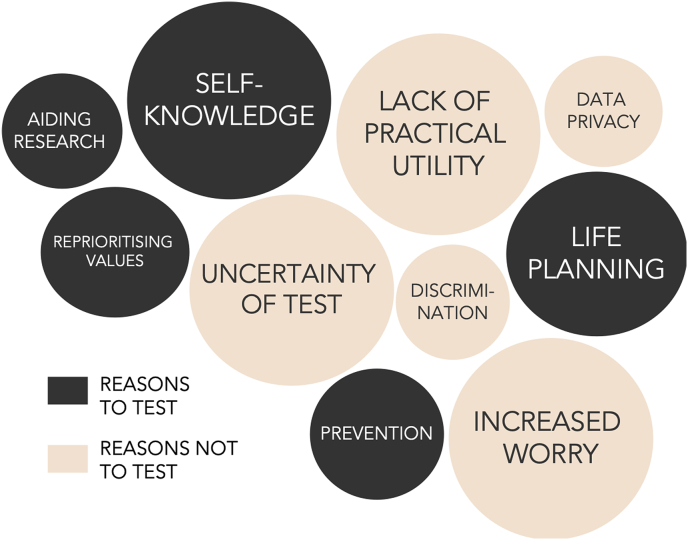
Key motivating factors to test or not to test for LOAD risk; larger circles represent more prominent themes.

Sixteen participants indicated they would not be interested in getting a test for LOAD, eleven indicated they would and three were ambivalent/neutral. The remaining participant had previously undergone testing through 23andMe. A summary of the key motivating factors to take or not the test is displayed in Fig. 3. It is important to note that participants elaborated on different factors that would motivate them to either test or not test, regardless of their personal opinion to test or not.

As illustrated, a core motivating factor for taking the test was self-knowledge regarding one's health (“You'd understand yourself a bit more”, Zak, 20; “If they find those genes in my DNA then I would want to know that they're there”, Chris, 23). Even though no participants reported a feeling of obligation to undergo testing for the benefit of family members, a recurrent theme was that knowing their risk status would help them plan ahead and ensure that their care would not place an emotional, financial or time burden on close others.

Because I think it'd be very good for life-planning, personally. So, if I've got a significant risk of developing Alzheimer's, I'd probably make sure I'm not a huge burden on my family, towards my latter age (Zak, 20).

A minority mentioned that learning their risk status would be a useful opportunity to reflect on what personally matters to them, and motivate them to re-prioritise personal values (“I would go out and do more stuff that I want to do, and try and have more fun, and … Make sure you enjoy each day, and each time, or moment, or whatever.” Priya, 26). Others mentioned the possibility of prevention as a motivating factor. For instance, Laura (20) mentioned that “it's always nice to know your risk of getting Alzheimer's and potentially doing something about it (…) start eating healthier and things like that.” Less common themes included a desire to advance Alzheimer's research (“you want to feel like you're helping the medical community develop knowledge in these really important issues” Tom, 19), following a medical advice and practical factors such as low cost and convenience.

Participants also elaborated on a number of factors that would deter them from getting tested. A core theme here was increased worry about the future if the test result was positive (“I feel like I don't really want that on my mind (…) If I knew that I did, that would stress me out”, Samantha, 20). A majority also mentioned the lack of practical utility (“I don't know, just like don't really see the point because there's no cure and there's nothing you can do about it.” Samantha, 20) and/or the uncertainty of the test (“the only thing that would make me consider getting the test is if it was 100%, no false positive, no false negatives. But that's never going to happen”, George, 20) as core concerns. Less common themes included practical issues such as high cost (“I can imagine some kind of cognitive testing that could be done remotely or cheaply but a DNA test seems very expensive to me”, Mel, 22), as well as concerns around data privacy and discrimination (“I suppose in terms of data protection and so on, it's a little bit worrying that all that genetic information is suddenly there available online and, I don't know, leaked to insurance companies”, Mabel, 20).

### 3.5 Vulnerability and the value of face-to-face support

A substantial majority expressed a personal preference for a clinical or research setting over the online option, if they were to take a test for LOAD risk. When discussing their choice, participants focused on the availability of face-to-face interactions with experts to obtain what they considered to be reliable information, advice, and emotional support. For example, Sharon (20 years old) expressed that “if you have a problem anyway they're equipped to deal with whatever”, and Amy (21 years old) claimed that “if someone else was there they'd be able to calm me down and offer me some sort of comfort.” Participants also expressed greater trust in face-to-face services. For example, some mentioned that a face-to-face, clinical service would make them feel more control over the process and the way their information is handled. In one participant's words, “I think it would be nice to have the reassurance that you know where it [the information] is going” Maya, 19).

Overall, participants emphasised the vulnerable position of someone seeking risk information about future illness. For example, Lea (18 years old) described getting a test as a “scary and confusing time”; and argued that “if you're doing the test, even regardless of the result, you're quite emotionally vulnerable”. Similarly, when reflecting upon her reasons not to test, Mary (22 years old) mentioned: “it makes me very scared that I will have it because watching my grandmother and her deterioration (…) I would hate … I'd just hate to live like that.”

## 4 Discussion

This is the first study to examine moral attitudes and motivations with regards to DTC genetic testing for LOAD in a sample of young people whose grandparents were diagnosed with the condition. Most participants were in favour of DTC genetic testing for LOAD, arguing that individuals have the right to know and access information about themselves. However, they also expressed concerns over the lack of assistance offered by these services. In particular, many argued for the need to protect children and adolescents from potential harms of LOAD testing. Participants' motivations for testing were largely grounded on a desire for self-knowledge regarding one's health; however, most did not wish to get tested, primarily due to the distress a high-risk result might cause. The Internet was not generally perceived as a well-supported, trustworthy space for genetic screening, and a substantial majority preferred clinical face-to-face services over the online option.

A core argument against genetic testing for APOE is that the risk information provided is not clinically relevant (Goldman et al., 2011). The lack of medical actionability, combined with potential risks such as anxiety, discrimination and stigma, drove the argument that these tests are not permissible (Evans, 2001). We found, however, that genetic testing for LOAD includes personal utilities that go beyond clinical benefit, such as self-knowledge regarding one's health and the opportunity to reflect on and re-prioritise personal values. These reasons differed from what has been reported by first-degree relatives of people with Alzheimer's, who were primarily motivated by worries about memory loss and the possibility to prepare family members for LOAD (Cutler and Hodgson, 2003). The range of motivating factors cited by our research participants suggest, as put forward by Vayena (2015), that DTC tests offer “plural utilities” with independent significance, which must be considered when judging their permissibility.

This opens up a discussion for health care professionals, in particular clinical genetic counsellors, when advising on predictive genetic testing in a clinical setting. Clinical genetic counsellors report that factors other than the patient's best medical interests are largely beyond the scope of their knowledge (Fenwick et al., 2017). With a more holistic knowledge of nonmedical factors that matter to young people, and an understanding of general attitudes towards predictive tests for LOAD, counsellors will be able to facilitate a more confident and balanced discussion with young people. In our study, many participants anticipated a positive risk result to be distressing. Counsellors should explore such feelings and concerns with parents and young people who used (or wish to use) DTC services to learn about LOAD risk.

Even though participants generally argued for liberal regulatory schemes for DTC LOAD testing, in line with recent ethical accounts (Vayena, 2015; Bonython and Arnold, 2018), most were unwilling to personally take a test. Furthermore, online DTC services were perceived to be at odds with what they considered to be the serious, medical nature of the procedure. This perception of DTC services might stem from these companies’ marketing strategy, which typically frames the service as a “fun consumer product” (Felzmann, 2015). Importantly, most participants valued support in the context of genetic testing and understood that to mean face-to-face interactions with health providers. No participants mentioned online information sheets, which are common practice in the DTC testing arena, as a preferred option.

Given that the Internet is increasingly recognised by young people as an important site to obtain health information and advice (Borzekowski and Rickert, 2001) it is important that DTC companies provide appropriate standards of information and support to their young customers. Effective protocols for communicating LOAD risk—online and in-person—should be developed and tested with minors as well as parents who request tests on behalf of their children, as it has been done for adults (Roberts et al., 2012). These protocols must take into account the emotive nature of the test for those with a family history of the disease. This is not only relevant for DTC companies—recent evidence suggests that health professionals may not be ready to provide feedback on such tests and sometimes catastrophise the meaning of a positive APOE ε4-positive result (Zallen, 2018). Appropriate training of health professionals is key, as the demand for guidance and support might increase as DTC services grow in popularity.

Participants' attitudes towards LOAD testing, particularly their preference for face-to-face services, might be due to their familial circumstances. All participants had a grandparent with LOAD and many had close contact with them, which arguably made them acutely aware of the seriousness and debilitating nature of the condition. Several studies suggest that Alzheimer's disease has a large impact on family dynamics and elicits stress and anxiety in adolescent grandchildren (Szinovacz, 2003). It is, therefore, unsurprising that the prospect of being at genetic risk was described as emotionally triggering and a potential burden for our study participants. The vulnerability of grandchildren who have these first-hand experiences is something that must be taken seriously by health professionals, DTC companies, policymakers and other relevant stakeholders, if this test is offered to young people. Decision aids such as the one developed Ekstract and colleagues (Ekstract et al., 2017), if validated for children and young people, might provide tailored, entry-point educational assistance to those considering APOE testing. In the clinic, healthcare professionals must include young people in discussions about LOAD risk in the family and the optimal time of testing when parents request predictive genetic testing of children. These initiatives are important, especially given that young people's capabilities and vulnerabilities are variable and not necessarily tied to (often arguably arbitrary) age thresholds for consent (Noroozi et al., 2018).

Finally, participants had mixed opinions when it comes to testing children with parental consent: while some argued, in concert with current recommendations (Borry et al., 2006), that testing is unnecessary and potentially harmful before the age of consent, others argued for the parents' right to decide. It is worth noting that even though risks and benefits to a child's future autonomy is the core ethical concern discussed in this literature (Borry et al., 2014; Robertson and Savulescu, 2001), this was rarely cited by the present participants. Rather, their objections were mostly grounded on potential adverse psychological effects of thinking about risk for LOAD and receiving that information at an early age. It is crucial that this impact is empirically tested in future studies with children and adolescents under the age of 18. Indeed, the current literature involving adult participants has already documented adverse reactions when risk information is disclosed online (Zallen, 2018), but not in-person or through the phone (Christensen et al., 2018).

Young people's concerns around potential deleterious effects of learning about one's risk for minors clash with DTC services' current practices: a parent's consent is all that is required from DTC companies to test one's child, with little robust screening or guidance. This discrepancy points to the need for more communication among DTC services, young people and professional medical bodies. Better regulation of DTC services available to minors is also warranted, to ensure appropriate safeguards are in place.

### 4.1 Limitations

Several limitations of the study must be acknowledged. First, the questions posed regarding predictive genetic testing were all hypothetical. Although use of hypothetical scenarios is a standard methodology in bioethics, the results only approximate real world conditions. Therefore, it is unclear whether the motivations and arguments would match those that actually are the basis for making a real decision. Discrepancies have been previously reported in research on predictive genetic testing for Huntington disease, where actual uptake was significantly lower than expressed interest (Quaid et al., 1989). Our use of a realistic advertisement in this study possibly helped close the distance between fictional and real world conditions, in so far as participants were invited to imagine themselves in a real-life scenario. Role-play has been shown to elicit authentic reflection around one's motivations, values and beliefs (Pavarini et al., 2020).

Another potential limitation stems from having used a video to communicate basic scientific facts regarding LOAD, which might have biased participants' perspectives. The video was, however, a valuable resource for participants to feel more confident in their knowledge and make informed decisions during the interview. We also assumed that in a real-life situation, participants might access some background information via DTC companies' pages or other platforms (e.g. YouTube) before requesting their test. However, investigating young people's motivations and perspectives in the absence of any background knowledge is critical for future research, as this might impact the results. In a recent study, it was found that women's interest in genetic testing for breast cancer was largely grounded on unrealistic beliefs about its predictive value and prevention prospects (Press et al., 2001), and decreased when accurate information was available. Future research should investigate if a similar pattern applies to LOAD test, to guide educational efforts around genetic testing.

A few limitations regarding this study's sample are also worth mentioning. Our sample was small and all participants were UK residents, where healthcare is offered through the public health system, and not typically seen as a consumer good (for similar results in Australia see ref (Critchley et al., 2015).). The sample was also biased toward university students from economically developed areas. Future studies should investigate whether our results hold in larger and more diverse samples of young people, including those without experiential knowledge of the condition. Furthermore, since most participants in our sample had reached the age of majority, future research should target children and adolescents under the age of 18, whose attitudes and perspectives are critical to the debate around consent.

Finally, all interviews were conducted face-to-face by university researchers. It is possible that the status of the interviewers influenced participants’ responses e.g., prompting them to think of the scientific validity of predictive testing results. The personal, in-depth nature of the interviews might also have favoured the emergence of certain themes e.g., vulnerability and support, which may not have been identified in a short survey. Future research using different methods such as peer interviewing, focus group and quantitative surveying will help ascertain the consistency of the results.

## 5 Conclusion

As many people purchased DTC genetic tests in 2018 as in all previous years combined (Regalado, 1288). Even though most young people are still not aware of these services (Mackert et al., 2012), it is likely that with persistent advertisement and growing popularity, these tests will become much more accessible to young people. Echoing previous research (Godino et al., 2016), young people interviewed were highly motivated to engage in ethically-relevant discussions about genetic testing, and offered valuable, well-reasoned insights. These insights resonated with, but did not mirror, professional guidelines and the current ethics literature; these dissonances must be bridged to provide optimum care for young people. Alongside the research presented in this paper, more research with young people is needed to enrich and inform ethical thinking and guide the development of regulations that strike the right balance between their right for autonomy and protection in the age of genomics. With increased understanding of young people's attitudes and motivations towards LOAD testing, clinical genetic counsellors can thus be placed in a better position to explore the idea of predictive genetic testing with young people and their families, using a holistic approach to make a decision based on the patient's best interests.

## Footnotes

1 Sawyer et al. (2020)'s definition of adolescence spans from age 10–24 and young adulthood spans from 25-29. The great majority of young people interviewed in the study were aged 19–23 so we consider the paper to be mainly relevant to adolescents. However, we recognise that we also have a few young adults in the sample (as per the Sawyer's definition), and because these age thresholds are ambiguous and variably defined, we will generally adopt the term ‘young people’ throughout this paper.
